# Large genomic deletion linked to field-evolved resistance to Cry1F corn in fall armyworm (*Spodoptera frugiperda*) from Florida

**DOI:** 10.1038/s41598-022-17603-3

**Published:** 2022-08-09

**Authors:** R. Banerjee, C. P. De Bortoli, F. Huang, K. Lamour, R. Meagher, D. Buntin, X. Ni, F. P. F. Reay-Jones, S. Stewart, Juan Luis Jurat-Fuentes

**Affiliations:** 1 Department of Entomology and Plant Pathology, University of Tennessee, Knoxville, TN 37996 USA; 2 Genome Science and Technology Graduate Program, University of Tennessee, Knoxville, TN 37996 USA; 3 Department of Entomology, Louisiana State University Agricultural Center, Baton Rouge, LA 70803 USA; 4 USDA-ARS Center for Medical, Agricultural and Veterinary Entomology, Insect Behavior and Biocontrol Research Unit, Gainesville, FL 32608 USA; 5 Department of Entomology, University of Georgia, Griffin, GA 30223 USA; 6 USDA-ARS Crop Genetics and Breeding Research Unit, Tifton, GA 31793 USA; 7 Plant and Environmental Sciences Department, Clemson University, Pee Dee Research and Education Center, Florence, SC USA

**Keywords:** Entomology, Genetic linkage study

## Abstract

The fall armyworm (*Spodoptera frugiperda*) is a highly polyphagous lepidopteran pest of relevant food and fiber staple crops. In the Americas, transgenic corn and cotton producing insecticidal proteins from the bacterium *Bacillus thuringiensis* (Bt) have controlled and reduced the damage caused by *S. frugiperda*. However, cases of field-evolved *S. frugiperda* resistance to Bt corn producing the Cry1F insecticidal protein have been documented in North and South America. When characterized, field resistance to Cry1F is linked to insertions and mutations resulting in a modified or truncated ABC transporter subfamily C2 (*SfABCC2*) protein that serves as Cry1F receptor in susceptible *S. frugiperda*. In this work, we present detection of a large genomic deletion (~ 8 kb) affecting the *SfABCC2* and an ABC transporter gene subfamily 3 –like gene (*SfABCC3*) as linked to resistance to Cry1F corn in a *S*. *frugiperda* strain from Florida (FL39). Monitoring for this genomic deletion using a discriminatory PCR reaction in field-collected *S. frugiperda* moths detected individuals carrying this allele in Florida, but not in surrounding states. This is the first report of a large genomic deletion being involved in resistance to a Bt insecticidal protein.

## Introduction

Larvae of the fall armyworm, *Spodoptera frugiperda* (J. E. Smith), are a relevant pest of staple food and fiber crops, including corn, soybean, sorghum, cotton, rice, and vegetables^1^. This insect is native to the American tropics, yet in the last 5 years *S. frugiperda* has been reported as a destructive invasive pest in sub-Saharan Africa, India, southeastern Asia, and Australia^2–5^. Depending on the level of infestation, corn yield losses from damage by *S. frugiperda* larvae can reach 73% in its native range^6^, while estimates in 12 African countries project up to US$ 6.3 billion revenue loss per year only from damage to corn^7^.

In the Western Hemisphere, transgenic corn and cotton producing Cry1F and other insecticidal proteins from the bacterium *Bacillus thuringiensis* (*Bt*) control larvae of *S. frugiperda*. However, *S. frugiperda* populations in Puerto Rico and the continental USA (Florida and North Carolina)^8,9^, Brazil^10^, and Argentina^11^ have developed practical resistance to Cry1F corn (event TC1507). Furthermore, cross-resistance to Cry1Ab and Cry1A.105 insecticidal proteins that are also produced by transgenic corn was observed in Cry1F-resistant *S. frugiperda*^12–14^. The mechanism of resistance to Cry1F in populations from Puerto Rico^13,15^ and Brazil^16^ involved indels and mutations in an ABC transporter subfamily C2 (*SfABCC2*) gene encoding a SfABCC2 protein that serves as Cry1F and Cry1A toxin receptor.

Predicted migratory movement of *S. frugiperda* from the Caribbean^17^ suggested a route for spread of Cry1F resistance alleles from Puerto Rico to overwintering *S. frugiperda* populations in Florida. In agreement with this hypothesis, results from complementation assays established that Cry1F resistance in the FL39 strain from Florida^9^ involved the same genetic locus as a resistant strain from Puerto Rico^18^. However, extensive genotyping efforts for the predominant Cry1F resistance allele in Puerto Rico (*SfABCC2mut*) failed to detect this allele in *S. frugiperda* populations from the continental USA^13,15^.

The goal of this project was to identify the allele responsible for Cry1F resistance in the FL39 strain and estimate its frequency in field populations of *S. frugiperda*. Taqman genotyping confirmed that the SfABCC2mut was not present in the FL39 strain or in field samples from Florida or nearby states Georgia and Alabama. Sequencing of the full length *SfABCC2* cDNA from FL39 larvae identified an aberrant transcript containing a premature stop codon predicted to result in a heavily truncated SfABCC2 protein. Results from F_2_ tests and backcrosses confirmed linkage of this aberrant cDNA with resistance to Cry1F in FL39. At the genomic level, a deletion of approximately 8 kb affecting the *SfABCC2* locus explained generation of the aberrant *SfABCC2* transcript in FL39. Using a discriminatory PCR assay, we detected and estimated the frequency of this new allele, that we name *SfABCC2FL1R*, in *S. frugiperda* populations from the Southeastern USA.

## Results

### Screening for *SfABCC2mut* in populations of *S. frugiperda* in Florida, Georgia and Alabama

Based on a shared genetic resistance locus in *S. frugiperda* strains from Puerto Rico and Florida^18^, field-collected *S. frugiperda* moths from Florida were genotyped for the presence of the *SfABCC2mut* allele originally identified in Cry1F-resistant *S. frugiperda* from Puerto Rico^15^. Sampling locations included counties in the southern (Belle Glade, Palm Beach Co.; Miami, Miami-Dade Co.), and northern (Citra, Marion Co.; Hague, Alachua Co.; Williston, Levy Co.; Greenwood, Jackson Co.; Quincy, Leon Co.) parts of the state (Supplementary Fig. S1). Discriminatory Taqman tests^15^ did not detect the *SfABCC2mut* allele among the 2,970 genotyped samples. Additional genotyping efforts included *S. frugiperda* from neighboring states, Georgia (3 locations, 609 total samples) and Alabama (4 locations, 2,160 total samples), and also did not detect the *SfABCC2mut* allele.

### Alterations in the *SfABCC2* cDNA of FL39

**Figure 1:**
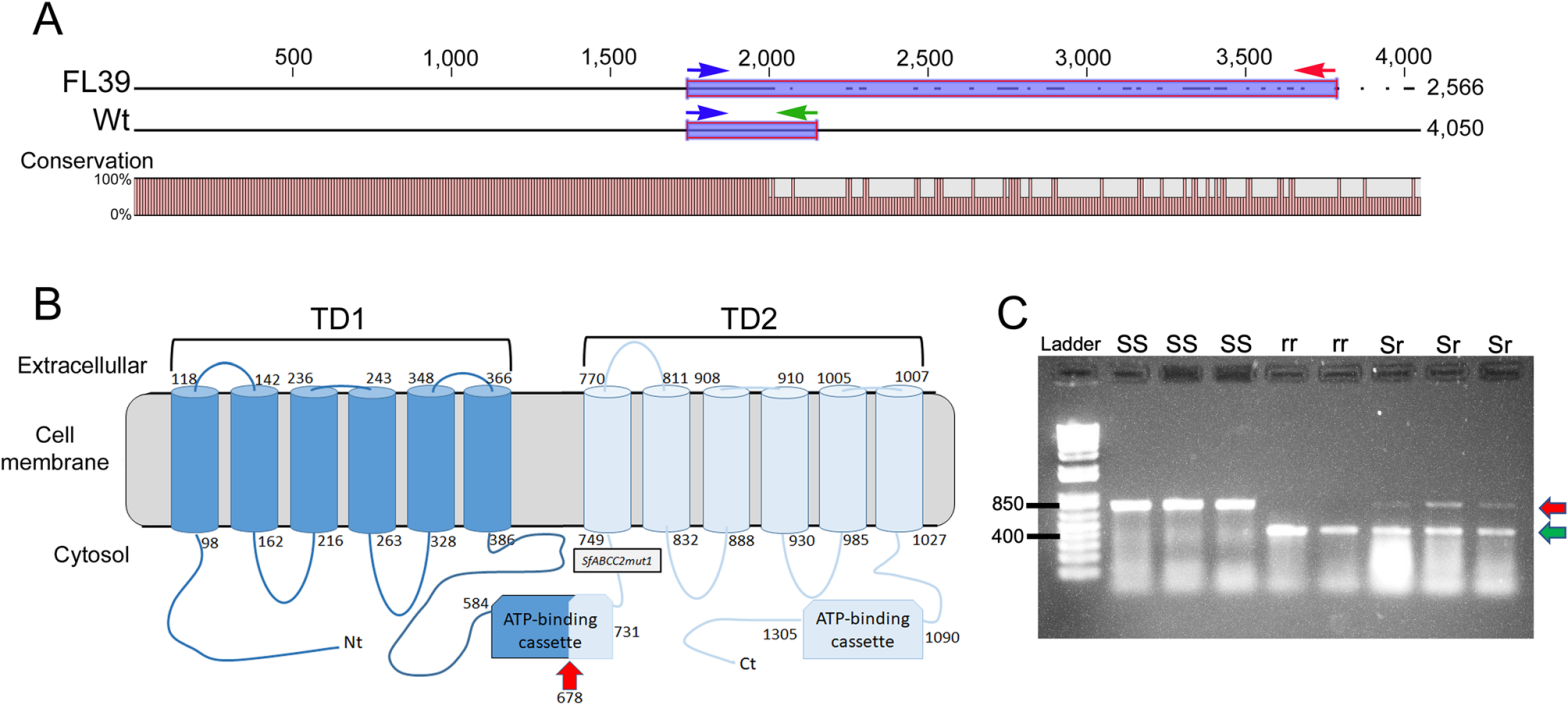
Comparison of the transcript and SfABCC2 protein in susceptible and Cry1F-resistant strains of *S. frugiperda*, and development of a discriminatory PCR reaction detecting the aberrant transcript in resistant larvae. (**A**) Alignment of the aberrant complementary DNA (cDNA) encoding the SfABCC2 protein amplified from midguts of the Cry1F-resistant FL39 strain (GenBank accession number OL791323) compared to the wild type (Wt) cDNA from a susceptible strain (Benzon, GenBank accession number KY489760) of *S. frugiperda*. Arrows indicate priming sites for a common forward (blue) primer and reverse primers specific to the Wt (green) or aberrant cDNA (red) used in a discriminative PCR reaction. (**B**) Diagram representing the predicted topology of the SfABCC2 protein in the cell membrane. Red arrow indicates location (amino acid residue) of predicted truncation in the aberrant SfABCC2 protein in strain FL39. Light blue coloring of the structure represents the region of the SfABCC2 protein that is predicted not to be produced in FL39 larvae. The truncation occurring in allele *SfABCC2mut* from Puerto Rico is indicated with a box. Numbers represent amino acid residues at the start of loops; TD = transmembrane domain. (**C**) Results from a discriminative PCR reaction with primers in (**A**) and cDNA from individual larvae. Shown on the left are molecular size marker positions for 400 and 850 bp. Amplicons of different size were detected for the aberrant *SfABCC2* cDNA in strain FL39 (r, 767 bp, red arrow) and the wild type *SfABCC2* cDNA (S, 411 bp, green arrow). Genotype (SS, Sr or rr) predicted from the observed amplicons is shown above each lane.

Primers amplifying the wild type *SfABCC2* cDNA sequence (GenBank accession number KY489760) failed to amplify a full-length cDNA from midguts of the FL39 strain. Consequently, we used RNA Ligase-Mediated (RLM)-Rapid Amplification of cDNA Ends (RACE) to amplify and clone the *SfABCC2* cDNA from FL39 larvae. Alignment of the FL39 amplicon to the reference *SfABCC2* cDNA sequence revealed an aberrant *SfABCC2* cDNA in FL39 after the first 2 kb (Fig. 1A). The aberrant *SfABCC2* cDNA was confirmed through sequencing in all examined FL39 individuals (*n* = 15) and based on being the first Cry1F resistance allele isolated in *S. frugiperda* from Florida, we named it as *SfABCC2FL1R*. Sixteen base pairs downstream from the start of divergence with the wild type, the *SfABCC2FL1R* cDNA sequence in FL39 had a predicted premature stop codon (Supplementary Fig. S2). When compared to the wild type, the predicted SfABCC2FL1R protein would include 679 amino acids and be a non-functional transporter containing only the first transmembrane domain and part of the first ATP binding cassette (Fig. 1B).

**Figure 2:**
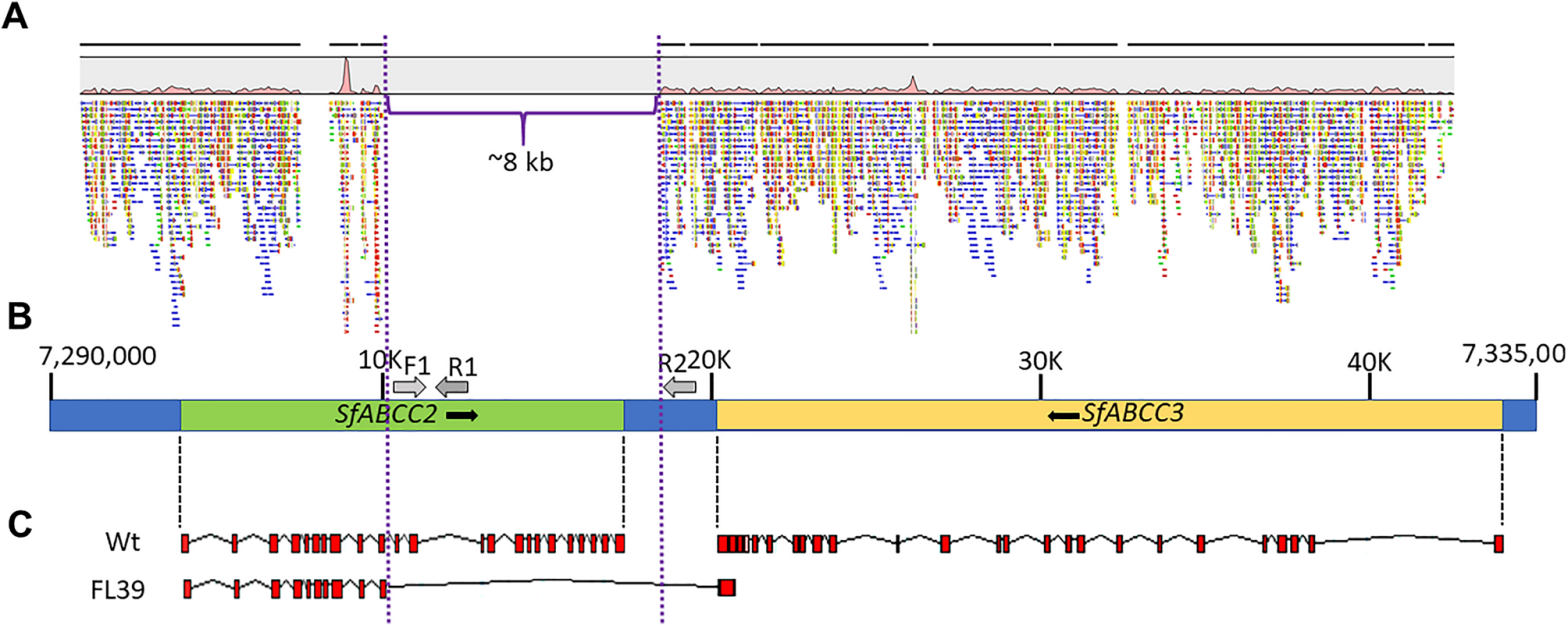
Diagram representing the genomic deletion in the *SfABCC2FL1R* allele. (**A**) Representative diagram of raw sequences mapping to the locus containing the *SfABCC2* and *SfABCC3* genes (Scaffold 33, nucleotides 7,290,000 to 7,335,000 in the *S. frugiperda* corn host strain genome v.6.0^38^) from sequencing the genome of larvae from the FL39 strain. The ~ 8 kb genomic region missing in the *SfABCC2FL1R* allele is delimited with purple dashed lines and open brace. (**B**) Diagram showing the *SfABCC2* and *SfABCC3* ORFs. Black arrows next to the gene names indicate that the genes are transcribed in opposite direction. Grey arrows represent the priming sites for a common primer (F1) and reverse primers specific to the wild type *SfABCC2* (R1) and *SfABCC2FLR1* (R2) alleles, respectively, that were used in discriminative PCR genotyping reactions. (**C**) Predicted exons (red) and introns (connecting lines) in the *SfABCC2* and *SfABCC3* genes in wild type (Wt), and Cry1F-resistant (FL39) strains. Dashed black lines indicate the relative location of the predicted transcripts for each gene in (**B**).

Analysis using BLASTn of the 3′ end in *SfABCC2FL1R* returned a highly significant match (E value = 4e − 130, 100% identity, 74% coverage) to the 3′ end of the *S. frugiperda ABCC3* transcript sequence (GenBank accession number KY646297.1). The *SfABCC3* gene locates to the opposite strand at roughly 2.7 kb downstream from the *SfABCC2* gene in Scaffold 33 from the *S. frugiperda* corn v6.0 genome. Alignment of *SfABCC2FL1R* with Scaffold 33 containing *SfABCC2* and *SfABCC3* genes revealed that the 3′ end of *SfABCC2FL1R* included a region in *SfABCC3* encompassing from 21 bp into the last (25th) exon to 62 bp into the 24th intronic region (Fig. 2A). A 73 bp intronic sequence between exons 24 and 25 was also present in *SfABCC2FL1R*. All these regions were missing from the wild type *SfABCC2* transcript (Fig. 2A).

### Linkage of *SfABCC2FL1R* with Cry1F resistance in FL39

Genotyping of F_2_ crosses and backcrosses was used to test linkage of the *SfABCC2FL1R* allele with resistance to Cry1F in the FL39 strain. The cDNA prepared from midgut RNA of individual larvae was used as template in a discriminative PCR reaction consisting of a common forward primer and two reverse primers specific to either the wild type or *SfABCC2FL1R* sequences. The reaction produced a 411 bp amplicon from cDNA of larvae from a susceptible *S. frugiperda* strain (Benzon) and a 767 bp amplicon from cDNA of the FL39 strain (Fig. 1A).

All the tested FL39 individuals (*n* = 14) were homozygous for *SfABCC2FL1R*, while the F_1_ larvae from Benzon × FL39 crosses (*n* = 15) were heterozygous (presented both amplicon bands, see Fig. 1C). Adults from the F_1_ generation were sib-mated or backcrossed to FL39 moths, and the resulting F_2_ generations were divided in two subfamilies that were fed with leaf material from corn event TC1507 producing Cry1F or its isoline. As expected from Mendelian transmission of a single recessive resistance allele, in the F_2_ subfamilies from sib-mating the genotype distribution did not significantly deviate from the expected 1:2:1 ratio for SS:Sr:rr genotypes (Table 1). Similarly, no significant deviation from the expected 1:1 Mendelian segregation ratio of genotypes (Sr:rr) was observed in the F_2_ backcross subfamily fed non-transgenic corn (Table 1). In contrast, all tested survivors from exposure to the TC1507 corn in the F_2_ from sib-mating were homozygous for *SfABCC2FL1R*, supporting cosegregation (Table 1). The genotype of survivors from exposure to TC1507 in the subfamily from the F_2_ backcross also showed significant deviation from the expected 1:1 genotype ratio (Table 1), further supporting linkage (cosegregation). The gender of the resistant moth in the backcross did not affect cosegregation results, in agreement with autosomal inheritance of resistance to Cry1F in the FL39 strain^18^.

**Table 1:** Cross and backcross F2 linkage tests for resistance to corn producing Cry1F (event TC1507) and the *SfABCC2FL1R* allele.

Strain/cross	Treatment	SS	SR	RR	Total (n)	P-value	χ^2^	Degrees of freedom
SS-TX		5	0	0	5			
FL39		0	0	5	5			
F1 (SS-TX × FL39)		0	15	0	15			
F2 (F1 × F1)	TC1507	0	0	20	20	< 0.0001*	60	2
F2 (F1 × F1)	Non-Bt	8	17	14	39	0.2883	2.487	2
F1 ♀ × FL 39 ♂	TC1507	0	3	13	16	0.0124*	6.25	1
F1 ♂ × FL 39 ♀	Non-Bt	0	10	10	20	1.0	0	1
F1 ♀ × FL 39 ♂	Non-Bt	0	4	4	8	1.0	0	1
F1 ♂ × FL 39 ♀	TC1507	0	7	18	25	0.0278*	4.840	1
F1 × FL39	Non-Bt	0	14	14	28	1.0	0	1
F1 × FL39	TC1507	0	10	31	41	0.0010*	10.756	1

The table presents the frequency of S (wild type *SfABCC2*) and r (*SfABCC2FL1R*) alleles in Cry1Fa susceptible (SS-TX) and resistant (FL39) strains of *S. frugiperda* and in F1 and F2 progenies from their crosses and backcrosses to the FL39 strain.

*Statistically significant difference when comparing observed and expected distribution of genotypes based on Mendelian transmission of a single recessive allele.

### Genomic characterization of the *SfABCC2FL1R* allele

Paired-end reads from sequencing the genomic DNA from 3 individuals of *S. frugiperda* strain FL39 were mapped to a roughly 45 kb fragment in Scaffold 33 encompassing the *SfABCC2* and *SfABCC3* genes. Approximately 2.9% (1,631,615) of the total reads were mapped to that fragment and were found covering the *SfABCC2* and *SfABCC3* gene sequences, except for a section of approximately 8 kb in the second half of *SfABCC2* to which no reads were mapped (Fig. 2A).

Amplification by PCR of a genomic DNA region encompassing the 8 kb deletion produced two amplicons of different size based on the larval *SfABCC2* genotype. Larvae homozygous for the genomic deletion produced a 426 bp amplicon, wild type homozygotes rendered a 1164 bp amplicon, while heterozygotes produced both bands (Fig. 2B). Sequencing and alignment of amplicons to the reference *SfABCC2* gene in Scaffold 33 confirmed the deletion of roughly 8 kb in larvae homozygous for the *SfABCC2FL1R* allele. Specifically, the deletion (8,054 bp) encompassed from 44 bp after the 10th exon to 829 bp after the predicted polyadenylation site for *SfABCC2* (Fig. 2C).

### Detection and estimation of *SfABCC2FL1R* allele frequency

The discriminative PCR assay described above used to amplify the wild type and *SfABCC2FL1R* allele from genomic DNA was used to genotype field-collected *S. frugiperda* adults. Moths were collected from four locations in Florida, two expected migratory flyways for *S. frugiperda* from Florida (Georgia, and South Carolina), and a location predicted to have low proportion of *S. frugiperda* from Florida (Jackson, Tennessee)^17^. Preliminary Taqman assays^15^ confirmed that none of the samples tested harbored the *SfABCC2mut* allele from Puerto Rico (data not shown). Genotyping results from the discriminatory PCR detected 5 individuals from Hague County that were heterozygous for *SfABCC2FL1R* (Table S1). No other positive samples were detected. These results suggest a frequency of 0.0036 for the *SfABCC2FL1R* allele in *S. frugiperda* samples from Florida (*n* = 1398), and 0.0060 among samples collected in Hague (Alachua Co., FL) (*n* = 835).

## Discussion

Whole genome sequencing and haplotype marker evidence suggest gene flow between *S. frugiperda* populations from Puerto Rico and Florida^19,20^. Detection of Cry1F resistance alleles in *S. frugiperda* from Florida^9^ shortly after reports of field-evolved resistance in Puerto Rico^8^ suggested migration of resistant moths into the continental USA. A disruptional mutation in the *SfABCC2* gene (*SfABCC2mut* allele) was linked to field-evolved resistance to corn producing Cry1F in Puerto Rico^13,15^. Results from complementation tests supported that field-evolved resistance in the FL39 strain from Florida also localized to the *SfABCC2* locus^18^. However, while the *SfABCC2mut* allele is highly frequent in Puerto Rico^20^, it has not been detected in *S. frugiperda* from the continental USA^13,15^, including genotyping results in the current work with samples from Florida and neighboring states (Georgia and Alabama). We present experimental evidence supporting a new allele we name *SfABCC2FL1R* that cosegregates with resistance in FL39. This allele involves deletion of a large (roughly 8 kb) *SfABCC2* genomic region predicted to encode an aberrant transcript resulting in a heavily truncated SfABCC2 protein, explaining the Cry1F-resistant phenotype. Heterozygous moths carrying the *SfABCC2FL1R* allele were detected at relative low frequency (0.0060) among field-collected *S. frugiperda* from Alachua County (Hague) in Florida.

Genotyping results with both F_2_ crosses and backcrosses provided strong statistical support for cosegregation of *SfABCC2FL1R* with resistance to corn event TC1507 producing Cry1F. An unexpected observation in these F_2_ linkage tests was the detection of *SfABCC2FL1R* heterozygotes among the backcrossed larvae surviving exposure to TC1507 corn. Similar survival of heterozygotes was reported in *SfABCC2mut* linkage tests using TC1507^15^. These observations could be explained by TC1507 not meeting the high toxin dose criteria for *S. frugiperda*^9,21^.

While miss-splicing resulting from deletions in ABCC2 genes have been linked with resistance to Cry1Ac in *Helicoverpa armigera* (Hübner)^22^ and *Plutella xylostella* (L.)^23^, the *SfABCC2FL1R* allele described here is the first example of a large ABCC2 gene deletion linked with resistance to a Cry protein. The predicted SfABCC2FL1R protein would functionally resemble the truncated SfABCC2mut protein produced in Cry1F-resistant *S. frugiperda* from Puerto Rico. Functional assays revealed loss of Cry1F binding and receptor ability in SfABCC2mut^15^. Therefore, resistance to Cry1F in the FL39 strain would also be related to lack of a functional SfABCC2 receptor for the Cry1F insecticidal protein.

In the *S. frugiperda* midgut Cry1F shares binding sites with Cry1A and Cry1A.105 insecticidal proteins^24^, and SfABCC2 is a shared receptor for Cry1F and Cry1A proteins^13,15^. Considering that Cry1A.105 is a Cry1A-Cry1F chimera, alterations in SfABCC2 would explain detection of cross-resistance between Cry1F and Cry1A.105 in *S. frugiperda*^12,13,25^. However, while the FL39 strain is highly resistant to Cry1F (> 270-fold), it presents low levels of cross-resistance (4.8-fold) to Cry1A.105^9^. The FL39 phenotype is similar to *S. frugiperda* collected in Puerto Rico, which displayed high resistance to Cry1F while remaining relatively susceptible to Cry1A.105^26^. This discrepancy may be suggestive of multiple resistance alleles present in strains resistant to both Cry1F and Cry1A.105^13^, or that Cry1A.105 and Cry1F may recognize physically close but distinct sites in SfABCC2 that are differentially affected by resistant mutations. Extracellular loops 2 and 4 in SfABCC2 are critical to binding and Cry1F toxicity^27^, yet there are no data available on the receptor regions for Cry1A.105 in SfABCC2.

Homozygotes carrying the *SfABCC2mut* or *SfABCC2FL1R* alleles are expected to produce a similar truncated SfABCC2 protein. However, relevant fitness costs were detected in the FL39 strain carrying the *SfABCC2FL1R* allele^28^ compared to a strain carrying the *SfABCC2mut* allele^29^. It is plausible that the large genomic deletion in *SfABCC2FL1R*, affecting *SfABCC2* and *SfABCC3* genes, may also affect regulation or expression of additional genes, leading to more relevant fitness costs. In fact, genetic knockouts of *SfABCC2* and *SfABCC3* are lethal {Jin, 2021 #22792} and the 3′ end of the genomic region deleted in *SfABCC2FL1R* includes a predicted ORF of unknown function (data not shown). More detailed annotation of the deleted genomic region in *SfABCC2FL1R* is necessary to test this hypothesis.

The distribution and frequency of resistance to Cry1F in populations of *S. frugiperda* in the continental USA is not well known. Estimates from an F_1_ screen in 2010–2011 using a Cry1F-resistant strain from Puerto Rico suggested total frequency of 0.1229 for resistance alleles in southern Florida counties^30^. Our PCR screen for the *SfABCC2FL1R* allele in Florida suggest lower frequency (0.0036) and lack of detection in Collier Co. where the FL39 strain was originally collected. One possibility to explain this difference is that resistance detected by Velez et al.^30,32^ may have involved an additional resistance mechanism or allele distinct from *SfABCC2FL1R*. We consider this to be improbable for several reasons. For instance, close proximity between Collier Co., where the FL39 was originally collected, and neighboring Hendry and Palm Beach Counties sampled by Velez et al.^30,32^, suggests the same allele involved could have spread in the area. While alternative resistance alleles may be present in the area, all cases of practical resistance to transgenic Bt crops in *S. frugiperda* described at the molecular level to date involved a mutation in the *SfABCC2* gene, as in the case of *SfABCC2FL1R*. Alternatively, it is possible that the frequency of *SfABCC2FL1R* has diminished in field populations since the screen by Velez et al.^30,32^, which would be supported by the fitness costs detected in the FL39 strain^28^. Temporal changes in available crop hosts and adoption of corn producing Cry1F could also affect distribution of the *SfABCC2FL1R* allele. Nevertheless, while we provide evidence for field detection, more exhaustive PCR screening efforts would be needed to more accurately determine the frequency and distribution of *SfABCC2FL1R* in Florida and southeastern USA.

Taken together, currently available data support that while there is gene flow between *S. frugiperda* from the Caribbean and continental USA^19,31^, practical resistance to Cry1F in North America emerged locally and not as a result of migratory dispersal. From a practical perspective, this finding indicates that for *S. frugiperda* evolution of resistance at one location may affect the risk of resistance at migratory destinations. Our current hypothesis is that resistant alleles affecting *SfABCC2*, the only relevant Cry1F resistance marker identified so far, are maintained at high relative frequency in *S. frugiperda* populations. This hypothesis is also in agreement with the lack of significant fitness costs commonly reported in insects carrying *SfABCC2* resistance alleles^29,32^. Exposure to corn producing Cry1A or Cry1F would thus result in relatively quick selection of individuals carrying these mutations and practical resistance, as observed in Puerto Rico and Brazil^33^. In agreement with this hypothesis, targeted *SfABCC2* gene sequencing in *S. frugiperda* from Puerto Rico confirmed high frequency of disruptive mutations predictive of Cry1F resistance^20^. In addition, multiple *SfABCC2* resistance alleles coexist in *S. frugiperda* populations^13,20^. Information on the frequency of putative *SfABCC2* resistance alleles in *S. frugiperda* populations where Cry1F corn is not yet deployed or where practical resistance is not reported is needed to further test the proposed hypothesis. The identification of additional *SfABCC2* resistance alleles will assist in tracing potential dispersal routes and improve resistance management strategies to delay the evolution and spread of resistance to insecticidal proteins produced by transgenic crops.

## Methods

### Insect strains and samples

Insect populations were maintained in the laboratory as described elsewhere^18^. The SS-TX strain of *S. frugiperda* was collected from non-Bt corn near Weslaco (Texas) in 2013 and is susceptible to Cry1A, Cry1F and Vip3Aa toxins^9,34^. The FL39 strain was generated from collections in non-Bt corn in Collier County (Florida) in 2011, and was > 270-fold resistant to Cry1F, but susceptible to Cry2Ab2 and Vip3Aa when compared to the SS-TX strain^9^. After its establishment, the FL39 strain was maintained in the laboratory for approximately 1 year before midgut samples were extracted and used in the current study. During this period, the FL39 strain was back-crossed twice to SS-TX and the resistance re-selected on Cry1F maize leaf tissue for three additional generations with a selection pressure of > 90% in each generation. A published study documented that high levels of Cry1F resistance were maintained after backcrossing and reselections^18^. Midguts extracted from SS-TX and the backcrossed-and-reselected resistant FL-39 were used in the current study.

Adult *S. frugiperda* (moths) were captured using sex pheromone baited traps^35^ at sites near corn plantings to increase probability of capturing corn host strain individuals. The collected specimens were visually inspected and identified as *S. frugiperda* by morphology^36,37^ and stored at − 20 °C until required for analysis. When needed, we refer to samples using a naming protocol described previously^31^, which includes the first three letters (USA) representing the country of origin, the next two letters representing the first two letters of the state of origin (AL for Alabama, GA for Georgia, FL for Florida, SC for South Carolina, TN for Tennessee), and a number differentiating samples from the same location.

### Detection of *SfABCC2mut* allele

Moths collected from pheromone traps was used in a discriminatory Taqman assay for detection of the *SfABCC2mut* allele, as previously described^15^. Genomic DNA (gDNA) was isolated from head/thorax or legs of moths using the Pure Link Genomic DNA kit (Invitrogen), following the manufacturer’s protocol, and then quantified using a Nanodrop spectrophotometer. Detection of the *SfABCC2mut* allele using Taqman probes was performed in 10 µl (final volume) reactions including 10–20 ng of gDNA as template, a VIC-labeled probe specific to the *SfABCC2mut* allele, a FAM-labeled probe specific to the wild type *SfABCC2* allele, and forward and reverse primers specific to amplify a 59 bp fragment in the *SfABCC2* gene. Reactions used parameters previously described^15^ and were monitored using a Quant studio 6 Real Time PCR instrument (Applied Biosystems). Post-amplification intensity of the fluorescent probes measured in the post-read stage was used by the software to generate an allelic discrimination plot.

### Amplification of *SfABCC2* in FL39

Total RNA was purified from midguts of 4th instar larvae from the FL39 strain using the TRIzol reagent (Invitrogen), following manufacturer’s instructions. A cDNA template was then prepared from 2 µg of the total RNA with random hexamer primers using the High Capacity cDNA Reverse Transcription kit (Invitrogen), according to the manufacturer’s instructions. The cDNAs prepared were first used in a touch-down PCR protocol using Q5 High-Fidelity master mix (New England Biolabs) and primers designed to the 5′ and 3′ ends of the *SfABCC2* cDNA sequence (GenBank accession number KY489760). Specific PCR conditions included initial denaturation at 98 °C for 30 s, then a first step of 15 cycles of denaturation at 98 °C for 10 s, annealing for 30 s at 62 °C and decreasing the temperature 1 °C per cycle, and extension for 2 min at 72 °C, followed by 30 cycles in which the annealing temperature was kept at 47 °C, and then a final extension of 7 min at 72 °C.

Amplification of the 3′ end of the *SfABCC2* cDNA in the FL39 strain was performed using RNA-ligase mediated rapid amplification of cDNA ends (RLM-RACE) with the FirstChoice RLM-RACE kit (Invitrogen), following the manufacturer’s protocol. A gene specific forward primer (5′ ATACCGCGGCAAATGGCAATG 3′) was designed from the known *SfABCC2* sequence and used with the 3′ RACE Outer reverse primer provided with the kit. Thermal cycling conditions for amplification included initial denaturation at 98 °C for 30 s followed by 30 cycles of denaturation at 98 °C for 10 s, annealing at 55 °C for 30 s, and extension at 72 °C for 2 min, and finished with incubation at 72 °C for 5 min. Analysis by agarose gel electrophoresis detected multiple amplicons ranging from 200 to 2500 bp, and 1 μl of the sample was used as template for a second RLM-RACE amplification with the same cycling conditions but using a different forward gene specific primer (5′ GCTTATGAGTGAAATTATCAACGG 3′) and the 3′ RACE Inner reverse primer from the kit. Analysis by agarose gel electrophoresis detected 767 bp and ~ 2500 bp amplicons. The longest amplicon (~ 2500 bp) was purified from agarose gels after electrophoresis using a Gel Purification kit (Invitrogen) and sequenced by primer walking at the University of Tennessee Sequencing Facility (Knoxville, TN). The partial sequences obtained were aligned and assembled into a cDNA sequence of 2566 bp containing an ORF of 2037 bp and a poly (A) tail at the 3′ UTR, which is available at GenBank under accession number OL791323.

### Gene and protein sequence analyses

A consensus *SfABCC2* cDNA sequence was generated after alignment of four published cDNAs (GenBank accession numbers KY489760.1, MG387043.1, MN399979.1, and KY646296.1) in CLC Genomics Workbench v21.0 (Qiagen). This consensus sequence was used as a query in a BLASTn search of the *S. frugiperda* corn host strain genome v.6.0^38^ in the LepidoDB site (https://bipaa.genouest.org/sp/spodoptera_frugiperda_pub/). The search identified Scaffold_33 as containing the *SfABCC2* gene. The FGENESH gene prediction platform^39^ was used to predict genes in a roughly 45 kb locus in Scaffold_33 (nucleotides 7,290,000 to 7,335,000) predicted to contain the *SfABCC2* (13,397 bp, two predicted transcripts with 23 and 24 exons each), and approximately 2 kb downstream in the lagging strand the *SfABCC3* gene (23,864 bp, two predicted transcripts of 24 and 25 exons each). This Scaffold_33_frag locus and the predicted *SfABCC2* and *SfABCC3* genes were used as reference for genomic comparisons.

The genomic DNA from a pool of three larvae from the FL39 population identified as belonging to the corn host strain was previously sequenced as sample USAFLr1 on an Illumina HiSeqX device running a 2 × 150 bp paired-end configuration, as reported elsewhere^31^. The dataset generated is available at the NCBI Sequence Read Archive (SRA) repository under SRR12044629, with associated metadata available under NCBI BioProject id PRJNA640063. Raw data were processed to remove low quality bases using the CLC Genomics Workbench v 20.0.4 (Qiagen, Aarhus, Denmark) trim function using default parameters before analysis. The cleaned paired reads (55,277,578) were then mapped using the “Maps Reads to Reference” tool in CLC Genomics Workbench v21.0.4 using default parameters to the reference *S. frugiperda* corn strain genome v6.0^38^. A total of 44,725,634 (81%) of FL39 reads mapped to the reference *S. frugiperda* v6.0 genome. The consensus Scaffold_33_frag sequence for the FL39 sample was derived by the mapping tool in CLC Genomics Workbench. All sequence alignments and translations were performed using default parameters in the sequence analysis tools in CLC Genomics Workbench v21.0.4. Protein membrane topology was predicted using TOPCONS^40^.

### Linkage tests

Genetic linkage with resistance to Cry1F was tested by genotyping backcross progenies, as described elsewhere^15^. Briefly, moths from the SS-TX strain (50) were mated with female moths (50) from the FL39 strain to generate a heterozygous F_1_ generation. Males of this F_1_ generation (50) were backcrossed with FL39 females (50) to generate an F_2_ generation. The F_1_ individuals were also sib-mated to generate an F2 cross. Neonates in the F_2_ cross and backcross were divided into two subfamilies; subfamily A was exposed to Cry1F corn (event TC1507) leaf tissue for 5 days, while subfamily B was reared for the same period on the non-transgenic isoline (2T777). Survivors were then put on artificial diet (beet armyworm diet, Frontier Agricultural Sciences, Newark, DE) until reaching fourth instar, when their midgut was dissected and flash frozen before storage at − 80 °C. Individual midguts were processed for total RNA purification and cDNA preparation as described elsewhere^15^.

The presence of the resistant allele was screened using a discriminatory PCR reaction with cDNA prepared from 2 µg of total RNA from individual midguts of 4th instar larvae, as described above for *SfABCC2* amplification. Three primers (170 nM each) were used in the reaction; a forward primer specific to the susceptible allele (5′-GGTGACTTGTCTCTGGTTGGGGA-3′), a reserve primer specific to the susceptible allele (5′-CTTGACTGACATCTTTGATATTCC-3′) and a second reverse primer specific to a region in the cDNA of resistant insects (5′-CATTTGATTGTGGCAGGTTGAT-3′), as detailed in Fig. 1A. Reactions were performed using cDNA (50 ng of total RNA equivalents) as template and Platinum Blue PCR SuperMix (Invitrogen, Waltham, MA) following manufacturer’s instructions. The thermal cycling conditions for the reaction included initial denaturation at 95 °C for 3 min followed by 30 cycles of denaturation at 95 °C for 30 s, annealing at 55 °C for 30 s and extension at 72 °C for 1 min. A final extension step at 72 °C for 5 min was added before holding at 4 °C until analysis by 1% agarose gel electrophoresis. Upon electrophoresis, amplicons were visualized under UV light in a gel imaging station (AlphaImager, Alpha Innotech, San Jose, CA). Two different size amplicons could be detected based on the presence of the susceptible (411 bp) and resistant (767 bp) alleles in a sample. Genetic linkage (cosegregation) was determined based on statistical deviation between observed and expected proportions of genotypes for Mendelian inheritance of an autosomal recessive trait evaluated using the Chi-square test, as described elsewhere^15^.

### Fall armyworm sample collection and processing

Moths were collected in five southeastern U.S. states (Fig. S1) that either reported Bt-corn resistant *S. frugiperda* (Florida)^9^, or are expected to receive a migratory *S. frugiperda* influx from Florida (Georgia, Tennessee and South Carolina)^17^. Sex pheromone baited traps^35^ were placed at sites near corn plantings to increase captures of C-strain. Collected specimens were stored at − 20 °C until required for analysis.

The legs, head and/or thorax of individual moths were dissected and used for genomic DNA (gDNA) purification using the Qiagen Tissuelyser (Qiagen, Germantown, MD) and subsequently the QIAcube HT and QIAamp 96 DNA kits, following manufacturer’s protocols. Purified genomic DNA was quantified using the Qubit dsDNA HS kit (Invitrogen, Waltham, MA) and stored at − 20 °C until used.

### Genotyping of field-collected *S. frugiperda* for *SfABCC2FL1R* allele

Pools of purified gDNA (10 ng total) from 50 individual field-collected *S. frugiperda* samples were used as template for genotyping in a discriminatory PCR reaction similar to the one used for genotyping cDNA experiments in linkage tests described above. When the *SfABCC2FL1R* allele was detected, the pool was subdivided in reactions with equal amounts of gDNA (10 ng total) from a pool of 10 individuals. When one of these pools were positive for *SfABCC2FL1R* we then tested the 10 samples individually. The PCR reactions included a forward primer annealing to an exon common to susceptible and resistant genotypes (5′-CTTGACGATCCTCTATCGGC-3′), and reverse primers specific to either an exon in the susceptible (5′-AGTAGTCGGTAGTGGTGGCA-3′) allele or an intronic region in the resistant (5′-CGCAGCAACCCATGATTGGA-3′) allele. Reactions (25 µl) included 12 µl of Platinum Superfi Master Mix (Invitrogen, Waltham, MA), 10 ng of gDNA, 0.4 µM of the forward primer and 0.2 µM of each reverse primer. The thermal cycling conditions were as described for genotyping of samples for genetic linkage tests. Amplicons were resolved on a 1% agarose gel and visualized in an AlphaImager imaging station. Samples were assigned a genotype based on the presence of different size amplicons for the susceptible (1,164 bp) and resistant (426 bp) alleles. Frequency of the *SfABCC2FL1R* allele was determined as described elsewhere^15^ using the Hardy–Weinberg equation with the formula: F = (2 × ObsAa + Obsaa)/[2 × (ObsAA + ObsAa + Obsaa)]; where “F” is the frequency of the “a” allele (*SfABCC2FL1R*) and “Obs” the observed frequency of each of the three possible genotypes for the allele.

## Supplementary Information

Supplementary Information.

## Data Availability

The datasets generated during and/or analyzed during the current study are available from the corresponding author on reasonable request.
