# Supplementary material for: Large genomic deletion linked to field-evolved resistance to Cry1F corn in fall armyworm (Spodoptera frugiperda) from Florida
Source: Sci Rep. 2022 Aug 9;12:13580. doi: 10.1038/s41598-022-17603-3 (PMC9363433; doi:10.1038/s41598-022-17603-3)
Supplement: Supplementary file 1 — Supplementary Information. [file 41598_2022_17603_MOESM1_ESM.pdf]

Supplementary Information

Table S1. Detection and frequency of *SfABCC2FL1R* allele in field-collected *S. frugiperda* adults based on a discriminative PCR genotyping reaction detecting the genomic deletion in the FL39 strain of *S. frugiperda*. All positive samples for *SfABCC2FL1R* were heterozygous.

State	Location (County)	*n*	*SfABCC2FL1R*
Florida	Belle Glade (Palm Beach)	351	0
	Citra (Marion)	16	0
	Williston (Levy)	52	0
	Hague (Alachua)	835	5
	Immokalee (Collier)	144	0
Georgia	Griffin (Spalding)	302	0
Tennessee	Jackson (Madison)	268	0
South Carolina	Florence (Florence)	191	0
TOTAL		2,159	5

Figure S1- Sampling locations for genotyping. Moths were collected at indicated locations and then used to detect the *SfABCC2mut* (black), *SfABCC2FL1R* (red), or both alleles (blue), as indicated.

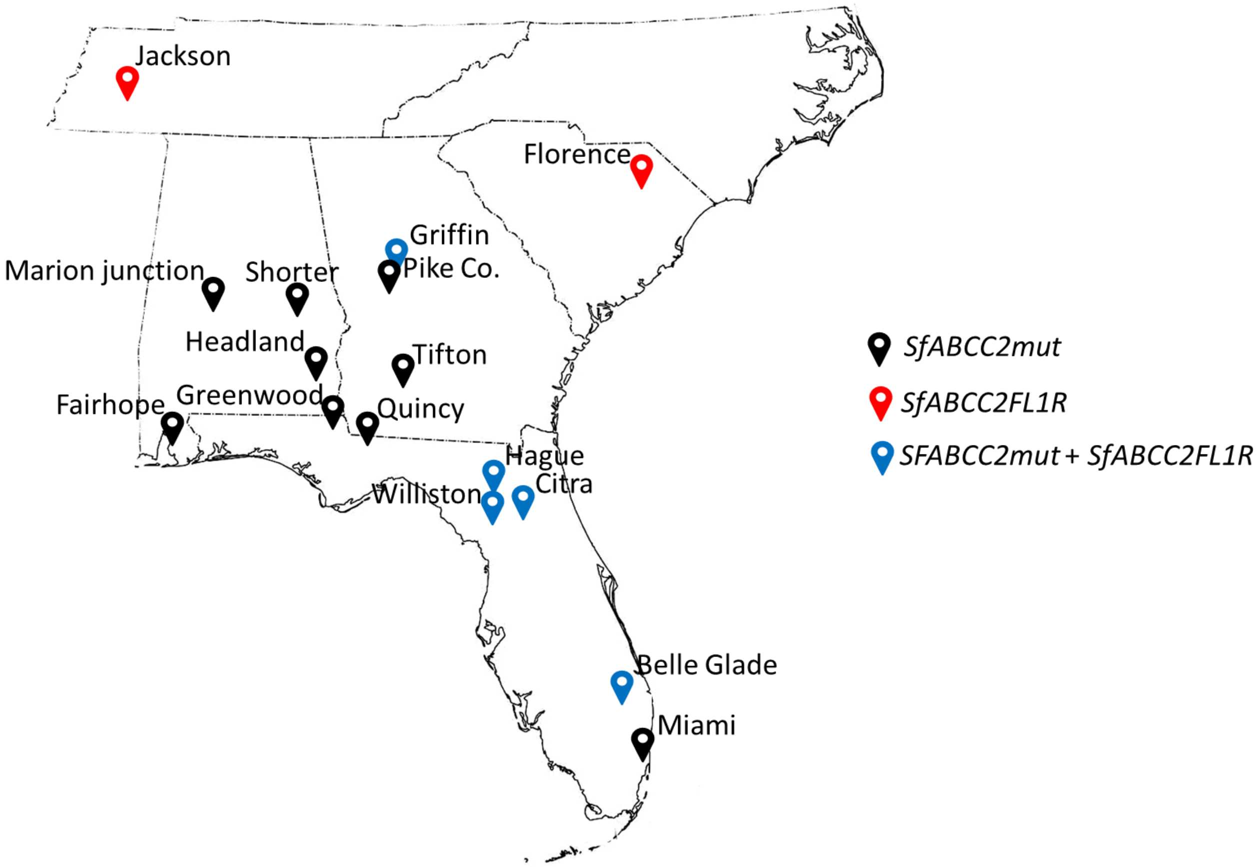

Figure S2- Alignment of the predicted protein sequences encoding by the wild type (*SfABCC2*) and *SfABCC2FL1R* alleles. Predicted transmembrane regions present in both proteins are in red and numbered. Asterisks represent conserved residues, a colon indicates different amino acids but belonging to groups of strongly similar properties, and a period indicates conservation between groups of weakly similar properties.

```
SfABCC2       MMDKSNKNTAANGNGGQRAGEPKERVRKKPNILSRIFVWWIFPVLITGNKRDVEEDDLIV  60
SfABCC2FL1R   MMDKSNKNTAANGNGGQRAGEPKERVRKKPNILSRIFVWWIFPVLITGNKRDVEEDDLIV  60
              ************************************************************

                                                               TM 1
SfABCC2       PSKKFNSERQGEYFERYWFEEVAIAEREDRDPSLWKAMRRAYWLQYMPGAIFVLLISGLR  120
SfABCC2FL1R   PSKKFNSERQGEYFERYWFEEVAIAEREDRDPSLWKAMRRAYWLQYMPGAIFVLLISGLR  120
              ************************************************************

                                         TM 2
SfABCC2       TAQPLLFSQLLSYWSVDSEMSQQDAGLYALAMLGINFITMMCTHHNNLFVMRFSMKVKIA  180
SfABCC2FL1R   TAQPLLFSQLLSYWSVDSEMSQQDAGLYALAMLGINFITMMCTHHNNLFVMRFSMKVKIA  180
              ************************************************************

                                                            TM 3
SfABCC2       ASSLLFRKLLRMSQVSVGDVAGGKLVNLLSNDVARFDYAFMFLHYLWVVPLQVGVVLYFV  240
SfABCC2FL1R   ASSLLFRKLLRMSQVSVGDVAGGKLVNLLSNDVARFDYAFMFLHYLWVVPLQVGVVLYFV  240
              ************************************************************

                            TM 4
SfABCC2       YDAAGWAPYVGLFGVIILIMPLQAGLTKLTGVVRRMTAKRTDKRIKLMSEIINGIQVIKM  300
SfABCC2FL1R   YDAAGWAPYVGLFGVIILIMPLQAGLTKLTGVVRRMTAKRTDKRIKLMSEIINGIQVIKM  300
              ************************************************************

                                                              TM 5
SfABCC2       YAWEKPFQLVVKAARAYEMSALRKSIFIRSMFLGFMLFTERSVMFLTVLTLALTGNMISA  360
SfABCC2FL1R   YAWEKPFQLVVKAARAYEMSALRKSIFIRSMFLGFMLFTERSVMFLTVLTLALTGNMISA  360
              ************************************************************

                              TM 6
SfABCC2       TLIYPIQQYFGIITMNVTLILPMAFASFSEMLISLERIQGFLLLDERSDIQITPKVVNGA  420
SfABCC2FL1R   TLIYPIQQYFGIITMNVTLILPMAFASFSEMLISLERIQGFLLLDERSDIQITPKVVNGA  420
              ************************************************************

SfABCC2       GSKLFNNSKKEGGLETGIVLPTKYSPTEANIARPMQDEPNMADYPVQLNKVNATWADLND  480
SfABCC2FL1R   GSKLFNNSKKEGGLETGIVLPTKYSPTEANIARPMQDEPNMADYPVQLNKVNATWADLND  480
              ************************************************************

SfABCC2       NKEMTLKNISLRVRKNKLCAVIGPVGSGKTSLLQLLLRELPVTSGNLSISGTVSYASQEP  540
SfABCC2FL1R   NKEMTLKNISLRVRKNKLCAVIGPVGSGKTSLLQLLLRELPVTSGNLSISGTVSYASQEP  540
              ************************************************************

SfABCC2       WLFPATVRENILFGLEYNVAKYKEVCKVCSLLPDFKQFPYGDLSLVGERGVSLSGGQRAR  600
SfABCC2FL1R   WLFPATVRENILFGLEYNVAKYKEVCKVCSLLPDFKQFPYGDLSLVGERGVSLSGGQRAR  600
              ************************************************************

SfABCC2       INLARAVYREADIYLLDDPLSAVDANVGRQLFDGCIKGYLSGKTCILVTHQIHYLKAADF  660
SfABCC2FL1R   INLARAVYREADIYLLDDPLSAVDANVGRQLFDGCIKGYLSGKTCILVTHQIHYLKAADF  660
              ************************************************************

SfABCC2       IVVLNEGSVENMG-SYDELMKTGTEFSMLLSDQASEGSDTDKKERPAMMRGISKMSVKSD  719
SfABCC2FL1R   IVVLNEITLEVSVFGYLE------------------------------------------  678
              ****** ::*    .* *

SfABCC2       DEEGEEKVQVLEAEERQSGSLKWDVLGRYMKSVNSWCMVVMAFLVLVITQGAATTTDYWL  779
SfABCC2FL1R   ------------------------------------------------------------  678

SfABCC2       SFWTNQVDGYIQTLPEGESPNPELNTQVGLLTTGQYLIVHGSVVLAIIILTQVRILSFVV  839
SfABCC2FL1R   ------------------------------------------------------------  678
```

```
SfABCC2      MTMRASENLHNTIYEKLIVAVMRFFDTNPSGRVLNRFSKDMGAMDELLPRSMLETVQMYL  899
SfABCC2FL1R  ------------------------------------------------------------  678

SfABCC2      SLASVLVLNAIALPWTLIPTTVLMFIFVFLLKWYINAAQAVKRLEGTTKSPVFGMINSTI  959
SfABCC2FL1R  ------------------------------------------------------------  678

SfABCC2      SGLSTIRSSNSQDRLLNSFDDAQNLHTSAFYTFLGGSTAFGLYLDTLCLIYLGIIMSIFI  1019
SfABCC2FL1R  ------------------------------------------------------------  678

SfABCC2      LGDFGELIPVGSVGLAVSQSMVLTMMLQMAAKFTADFLGQMTAVERVLEYTKLPTEENME  1079
SfABCC2FL1R  ------------------------------------------------------------  678

SfABCC2      TGPTTPPKGWPSAGEVTFSNVYLKYSPDDPPVLKDLNFAIKSGWKVGVVGRTGAGKSSLI  1139
SfABCC2FL1R  ------------------------------------------------------------  678

SfABCC2      SALFRLSDITGSIKIDGLDTQGIAKKLLRSKISIIPQEPVLFSASLRYNLDPFDNYNDED  1199
SfABCC2FL1R  ------------------------------------------------------------  678

SfABCC2      IWRALEQVELKESIPALDYKVSEGGTNFSMGQRQLVCLARAILRSNKILIMDEATANVDP  1259
SfABCC2FL1R  ------------------------------------------------------------  678

SfABCC2      QTDALIQKTIRKQFATCTVLTIAHRLNTIMDSDRVLVMDQGVAAEFDHPYILLSNPNSKF  1319
SfABCC2FL1R  ------------------------------------------------------------  678

SfABCC2      SSMVKETGDNMSRILFEVAKTKYESDSKTA      1349
SfABCC2FL1R  ------------------------------      678
```
